# Coding-Complete Genome Sequence of a Pollen-Associated Virus Belonging to the *Secoviridae* Family Recovered from a Japanese Apricot (*Prunus mume*) Metagenome Data Set

**DOI:** 10.1128/MRA.00881-19

**Published:** 2019-10-03

**Authors:** Andrea M. Fetters, Paul G. Cantalupo, Tia-Lynn Ashman, James M. Pipas

**Affiliations:** a Department of Biological Sciences, University of Pittsburgh, Pittsburgh, Pennsylvania, USA; DOE Joint Genome Institute

## Abstract

We report the coding-complete genome sequence of *Japanese apricot pollen-associated secovirus 1* (JAPSV1), a virus belonging to the *Secoviridae* family, recovered from Japanese apricot (Prunus mume) pollen that is closely related to *Peach leaf pitting-associated virus* (PLPAV). This discovery adds to the number of known pollen-associated viruses.

## ANNOUNCEMENT

The 2012 edition of *Virus Taxonomy* lists just under 900 species of plant viruses (1), yet far fewer are known to be pollen associated (2–4). The detection of such viruses in asymptomatic hosts and wild plants, however, is expected to accelerate viral discovery (1). *Secoviridae* is the fourth largest family of plant viruses, and viruses of this family are spread to plant hosts primarily by insects or nematodes (1). These viruses belong to the *Picornavirales* order and have a linear, positive-sense, single-stranded RNA (ssRNA) genome with two segments (5). The longer segment, RNA1, is approximately 6 to 8 kb and encodes all proteins necessary for cytoplasmic replication, and the shorter segment, RNA2, is 2 to 4 kb and encodes the capsid and movement proteins (5).

Pollen was collected by Akagi et al. (6) from anthers of flower buds in the balloon stage from trees in an experimental orchard at Kyoto University. They extracted the total RNA using the cold-phenol approach, and RNA sequencing (RNA-seq) libraries were constructed by the Japanese company TaKaRa using TruSeq RNA sample prep kits (Illumina). Akagi et al. (6) sequenced the RNA using the TruSeq SBS kit v3-HS on an Illumina HiSeq 2000 platform, which resulted in 35,162,899 paired-end informative Prunus mume cv. Kairyo-Uchida-Ume reads that were each 100 bp long (SRA run accession number DRR002284). We recovered *Japanese apricot pollen-associated secovirus 1* (JAPSV1) from this data set using Pickaxe, a viral discovery pipeline that detects known and novel viruses in sequence data (7, 8). Briefly, Pickaxe performs quality control on the raw reads and removes host reads (namely, *Prunus mume*, here) to obtain a set of nonhost reads. Nonhost reads are aligned to Viral RefSeq (ftp://ftp.ncbi.nlm.nih.gov/refseq/release/viral/) and are assembled with the CLC Assembly Cell (version 5; Qiagen), using default settings. The resulting contigs are annotated with a BLAST pipeline, as described previously (7), except that the BLASTX step was replaced with RAPSearch2 (9).

We identified two contigs, 6,133 bp and 2,704 bp, as RNA1 and RNA2, respectively, by BLASTN alignments, both with 93% identity to the genome segments of *Peach leaf pitting-associated virus* (PLPAV; RNA1, GenBank accession number MK460243; RNA2, accession number MK460244). RNA2 was increased by 690 bp through the use of an in-house script that found a substantial overlap region (94 bp) between the end of the 2,704-bp contig and the beginning of another contig, bringing the final length of RNA2 to 3,374 bp. The fact that the two contigs were not originally assembled is not surprising given the difficulty of *de novo* metagenomic assembly, since assembly programs assume the presence of one organism (10, 11). Aligning the nonhost reads to each genome segment resulted in greater than 99% coverage, with an average depth (reads/base) of 2,017 for RNA1 and 4,941 for RNA2. Both genome segments have 43% GC content. We found two open reading frames (ORFs) using the latest version of ORFfinder (https://www.ncbi.nlm.nih.gov/orffinder/), with default parameters that correspond to the *Secoviridae* genome. The longer segment of most *Secoviridae* genomes codes for helicase, proteinase, and polymerase proteins. Our conserved domain search of the longer ORF (1,920 amino acids) detected those proteins in the same sequence. Similarly, our conserved domain search of the shorter ORF (997 amino acids) detected a movement protein and two coat proteins in the same sequence as that of most *Secoviridae.* We searched the latest version of the Conserved Domain Database using the default parameters to annotate both RNA1 and RNA2 (12) (https://www.ncbi.nlm.nih.gov/Structure/cdd/wrpsb.cgi).

Due to the high percentage identity (98%) of the JAPSV1 polyproteins with those of PLPAV estimated using a BLASTP alignment, the similar GC content of the JAPSV1 polyproteins, and the fact that no other *Secoviridae* sequences were identified in the data set, we conclude that we detected a virus in the *Secoviridae* family that is closely related to PLPAV. Although JAPSV1 is closely related to PLPAV, its detection expands our knowledge of pollen-associated plant viruses and provides a starting point for considering how such viruses are spread, as well as plant virus host switching.

### Data availability.

This whole-genome shotgun project has been deposited in the European Nucleotide Archive under the accession numbers LR594708 and LR594709. The version described in this paper is the first version, and the coding-complete genome sequence of JAPSV1 is publicly available on the Pipas Lab website (http://pipaslab.webfactional.com/wp/wp-content/uploads/2019/07/JAPSV1.txt). The raw data in which we found JAPSV1 are available under SRA run number DRR002284, and the genome segment sequences of PLPAV are referenced by MK460243 and MK460244 in the National Center for Biotechnology Information.
